# Huaju Xiaoji Formula Regulates ERS-lncMGC/miRNA to Enhance the Renal Function of Hypertensive Diabetic Mice with Nephropathy

**DOI:** 10.1155/2024/6942156

**Published:** 2024-01-20

**Authors:** Zeng Zhang, Xiaodong Fu, Fengzhu Zhou, Duanchun Zhang, Yanqiu Xu, Zhaohua Fan, Shimei Wen, Yanting Shao, Zheng Yao, Yanming He

**Affiliations:** ^1^Department of Endocrinology, Yueyang Hospital of Integrated Traditional Chinese and Western Medicine, Shanghai University of Traditional Chinese Medicine, Shanghai 200437, China; ^2^Department of Integrated Traditional Chinese and Western Medicine, Huashan Hospital, Fudan University, Shanghai 200040, China

## Abstract

**Background:**

Better therapeutic drugs are required for treating hypertensive diabetic nephropathy. In our previous study, the Huaju Xiaoji (HJXJ) formula promoted the renal function of patients with diabetes and hypertensive nephropathy. In this study, we investigated the therapeutic effect and regulation mechanism of HJXJ in hypertensive diabetic mice with nephropathy.

**Methods:**

We constructed a mouse hypertensive diabetic nephropathy (HDN) model by treating mice with streptozotocin (STZ) and nomega-nitro-L-arginine methyl ester (LNAME). We also constructed a human glomerular mesangial cell (HGMC) model that was induced by high doses of sugar (30 mmol/mL) and TGF*β*1 (5 ng/mL). Pathological changes were evaluated by hematoxylin and eosin (H&E) staining, periodic acid Schiff (PAS) staining, and Masson staining. The fibrosis-related molecules (TGF*β*1, fibronectin, laminin, COL I, COL IV, *α*-SMA, and p-smad2/3) were detected by enzyme-linked immunosorbent assay (ELISA). The mRNA levels and protein expression of endoplasmic reticulum stress, fibrosis molecules, and their downstream molecules were assessed using qPCR and Western blotting assays.

**Results:**

Administering HJXJ promoted the renal function of HDN mice. HJXJ reduced the expression of ER stress makers (CHOP and GRP78) and lncMGC, miR379, miR494, miR495, miR377, CUGBP2, CPEB4, EDEM3, and ATF3 in HDN mice and model HGMCs. The positive control drugs (dapagliflozin and valsartan) also showed similar effects after treatment with HJXJ. Additionally, in model HGMCs, the overexpression of *CHOP* or *lncMGC* decreased the effects of HJXJ-M on the level of fibrosis molecules and downstream target molecules.

**Conclusion:**

In this study, we showed that the HJXJ formula may regulate ERS-lncMGC/miRNA to enhance renal function in hypertensive diabetic mice with nephropathy. This study may act as a reference for further investigating whether combining HJXJ with other drugs can enhance its therapeutic effect. The findings of this study might provide new insights into the clinical treatment of hypertensive diabetic nephropathy with HJXJ.

## 1. Introduction

Diabetes affects more than 425 million people around the world, and in 2045, the number of people with diabetes worldwide is estimated to be 693 million [1, 2]. The incidence of diabetic kidney disease (DKD) has increased considerably due to the prevalence of diabetes, and approximately 30–40% of diabetic patients develop DKD [3–5]. Most patients with DKD have diabetes mellitus and hypertension as the drivers of chronic kidney disease [6]. Early medical research showed that the first-line treatment for DKD, such as angiotensin II receptor blockers (ARB), could delay the progress of renal dysfunction, but the side effects of these drugs, such as hyperkalemia and angioedema, limited their application. Additionally, they could not decrease the rate of vascular events and mortality due to DKD [7, 8]. Therefore, therapeutic approaches that are more effective and have fewer side effects need to be developed to improve diabetic hypertensive nephropathy.

MicroRNAs (miRNAs) and long noncoding RNAs (lncRNAs) are non-protein-coding RNAs. They affect many cellular processes and are associated with the pathophysiology of diseases, such as diabetic kidney disease [9, 10]. A study shows that in the kidneys of diabetic mice, lncMGC is regulated by C/EBP homologous protein (CHOP) and endoplasmic reticulum stress- (ERS-) related transcription factor; also, in diabetic *Chop*^–/–^ mice, the levels of cluster microRNAs (miR379 cluster) and lncMGC decreased [11]. ER stress is prominent in various renal diseases, such as diabetic nephropathy, chronic kidney disease, and acute kidney injury [12]. Reduction of ERS may ameliorate renal fibrosis, and candesartan blocks ER stress-related apoptosis to restore renal integrity [13]. Therefore, ERS-lncMGC/miRNA may regulate the renal function in diabetic hypertensive nephropathy.

Traditional Chinese medicine may be an effective approach for treating diabetic hypertensive nephropathy. Randomized controlled clinical trials have confirmed that Chinese herbal formulae, consisting of different ingredients, are efficient and safe for DKD [2]. In our previous study, we found that administering the Yiqi Huaju formula improved the condition of patients with hypertension coupled with metabolic syndrome [14]. In salt-sensitive hypertension, the Yiqi Huaju formula also decreases arterial pressure by inhibiting the activation of the renin-angiotensin system [15]. Additionally, the Huaju Xiaoji (HJXJ) formula, which is being developed based on the Yiqi Huaju formula, has the potential to decrease urinary microalbumin, serum creatinine, and inflammation in patients with diabetes and hypertensive nephropathy [16]. However, the molecular mechanism and cellular processes underlying the action of the HJXJ formula need to be elucidated.

We hypothesized that the HJXJ formula may regulate ERS-lncMGC/miRNA to enhance renal function in hypertensive diabetic nephropathy. In this study, the therapeutic effect of HJXJ on hypertensive diabetic mice with nephropathy induced by streptozotocin (STZ) and nomega-nitro-L-arginine methyl ester (LNAME) was evaluated. The mechanism by which HJXJ regulates the ERS-lncMGC/miRNA pathway was also determined via *in vitro* and *in vivo* experiments.

## 2. Materials and Methods

### 2.1. HJXJ Extraction

For preparing HJXJ extracts, eight Chinese herbal medicines, including *Astragalus* (30 g), *Coptis chinensis* (10 g), puhuang (15 g), *Alisma* (15 g), mung bean (30 g), *Serissa japonica* (Thunb.) Thunb. (15 g), aconite (9 g), and *Centella asiatica* (15 g), were weighed. The raw medicinal materials were boiled, and the residue from the Chinese medicine was filtered using gauze. In total, 17.82 g of powder was obtained through vacuum freeze-drying. HJXJ was dissolved in water and administered to mice or cells.

### 2.2. Animal Models

Eight-week-old C57BL/6 mice (*n* = 50) were used in this study, six of which were randomly placed in the normal group (control, *n* = 6), and the remaining mice were used to construct hypertensive diabetic nephropathy (HDN) models [17]. Mice were intraperitoneally injected with STZ (50 mg/kg body weight) for five days [18]. One week after the end of the STZ treatment, the mice with high blood glucose levels (>4.5 mmol/L) were selected for subsequent experiments. The protocol followed was based on previous studies, with minor modifications. Briefly, the mice were treated with LNAME, which was added directly to their drinking water at a concentration of 2 g/L [19]. The drinking water was replaced daily for 16 weeks; each bottle contained approximately 150 mL of water. STZ-LNAME-induced mice (*n* = 36) were randomly divided into six groups (*n* = 6 mice in each group), which included model group, low-dose HJXJ (6.19 g/kg body weight) group (HJXJ-L group), medium-dose (12.38 g/kg body weight) HJXJ group (HJXJ-M group), high-dose (24.76 g/kg body weight) HJXJ group (HJXJ-H group), valsartan (ARB group) group, and dapagliflozin group (Dap group). After 12 weeks of intervention, the blood of mice was collected to detect transforming growth factor *β*1 (TGF*β*1), fibronectin, laminin, collagen (COL) I, and COL IV. After the mice were sacrificed, the renal tissues were fixed with 10% formalin solution, and then, the tissues were decalcified with 15% EDTA and embedded in paraffin. This study was conducted following the guidelines of the Declaration of Helsinki, and it was approved by the Ethics Committee of Yueyang Hospital of Integrated Traditional Chinese and Western Medicine, Shanghai University of Traditional Chinese Medicine (approval number: YYLAC-2021–125).

### 2.3. Biochemical Analysis

The levels of urinary albumin, serum creatinine, and blood urea nitrogen were measured using the corresponding assay kits following the manufacturer's instructions (Jiancheng Biological Technology Institute). Plasma triglyceride and total cholesterol levels were determined using the triglyceride content assay kit (BC0620, Solarbio) and the total cholesterol assay kit (A111–1–1, Nanjing Jiancheng Bioengineering Institute, Nanjing, China), respectively. The contents of high-density lipoprotein (HDL) cholesterol and low-density lipoprotein (LDL)/very-low-density lipoprotein (VLDL) cholesterol were detected using the HDL and LDL/VLDL cholesterol assay kit (ab65390, Abcam).

### 2.4. Tissue Staining for Pathological Analysis

The pathological examination of the tibia was performed by hematoxylin and eosin (H&E) staining, periodic acid Schiff (PAS) staining, and Masson staining. The paraffin-embedded blocks were cut into thin slices (4 *μ*m); then, the slices were dewaxed with xylene and rehydrated with alcohol. H&E staining was performed using the H&E staining kit (G1120, Solarbio, Beijing, China) following the manufacturer's instructions. The slices were stained with hematoxylin solution for 5 min and washed in an acetic acid solution for 30 s. The sections were counterstained with the eosin-phloxine solution for 2 min after they were rinsed with alcohol. For PAS staining, the rehydrated sections were treated with 0.5% periodic acid aqueous solution for 10 min. Then, the sections were stained with Schiff's solution for 30 min and washed thrice with sulfite solution. The sections were stained with hematoxylin for 3 min after they were washed with distilled water. For Masson staining, the rehydrated sections were treated with hematoxylin solution for 8 min, and the sections were stained with ponceau acid fuchsin solution for 10 min. The sections were counterstained with aniline blue solution for 5 min after they were treated with phosphomolybdic-phosphotungstic acid for 5 min. Then, the sections were dehydrated and mounted after differentiation in 1% acetic acid for 1 min. Finally, images were captured under an optical microscope to analyze the sections.

### 2.5. Enzyme-Linked Immunosorbent Assay (ELISA) Detection

The pretreatment media containing serum and human glomerular mesangial cells (HGMCs) were stored at −80°C until further analysis. The levels of COL I, COL IV, fibronectin, laminin, and TGF*β*1 in serum and HGMCs were evaluated using ELISA kits (CUSABIO, China). The serum was isolated from the blood of mice and left to clot for 2 h at room temperature or overnight at 4°C. Then, it was centrifuged for 15 min at 1,000 × *g*. The serum was removed and immediately analyzed or divided into aliquots and stored at –20°C or –80°C. After treatment, the supernatants of HGMCs from different groups were collected and centrifuged at 1,000 × *g* for 15 min at 4°C. Each sample was assayed in triplicate, and the experiment was repeated thrice to decrease the interassay and intra-assay coefficients of variation.

### 2.6. CCK-8 Assay

The HGMCs and human renal proximal tubular epithelial cells (HK-2) were cultured in a DMEM high-glucose medium containing 10% fetal bovine serum and 1% double-antibody (a mixture of penicillin and streptomycin) in an incubator at 37°C and 5% CO_2_. The cells in the logarithmic growth phase were digested by trypsin and added to 96-well plates. Different concentrations of HJXJ (0 mg/mL, 0.02 mg/mL, 0.05 mg/mL, 0.1 mg/mL, 0.2 mg/mL, 0.5 mg/mL, and 1 mg/mL) were used to treat cells. After 0, 24, 48, and 72 h of treatment with HJXJ, the cells were incubated in CCK-8 solutions (CP002, SAB) at 37°C for 1 h, and the absorbance of each well was measured at 450 nm.

### 2.7. HGMC Model

The HGMC cells in the logarithmic growth phase were digested by trypsin, counted under a microscope, and spread in six-well culture plates at a density of 300,000 cells per well. Then, 2 mL of a medium was added to each well, and the cells were cultured in an incubator at 37°C and 5% CO_2_. The cells were transfected when 60-70% fusion was recorded. High doses of sugar (30 mmol/mL) and TGF*β*1 (5 ng/mL) were used to treat HGMCs (model HGMC).

### 2.8. Cell Transfection

The transfer solution was prepared using the following methods. First, 250 *μ*L of the Opti-MEM medium (serum-free) was used as a control. *CHOP* and *lncMGC* were overexpressed to dissolve 5 *μ*L of *CHOP* and *lncMGC*-overexpression plasmids in 245 *μ*L of the Opti-MEM medium (serum-free). Then, 10 *μ*L of Lipofectamine 2000 was dissolved in 490 *μ*L of the Opti-MEM medium (serum-free), mixed thoroughly, and kept at room temperature for 5 min. The *CHOP*-overexpression transfer solution and *lncMGC*-overexpression transfer solution were mixed with one-half of the Lipofectamine 2000 transfer solution, respectively, and incubated for 20 min at room temperature. After the medium was discarded, the samples were rinsed once with sterile PBS, and then, 2 mL of a serum-free culture medium was added. Each tube complex was added to the corresponding culture medium, shaken well, and placed in an incubator at 37°C for 6 h. The serum-free transfer solution was replaced with the complete culture medium for further culture. Follow-up experiments were performed 48 h after transfection.

### 2.9. Quantitative Real-Time Polymerase Chain Reaction (RT-qPCR)

To extract RNA, the tissues and cells were treated with 1 mL of TRIzol reagent (Invitrogen), and the samples were treated with 200 *μ*L of chloroform and divided into the organic phase, the water phase containing RNA, and the phase containing protein and DNA. Then, the water phase was carefully transferred to a centrifuge tube, in which 600 *μ*L of isopropanol was added to precipitate RNA, and 1 mL of 75% absolute ethyl alcohol prepared with 75 *μ*L of absolute ethyl alcohol and 250 mL of diethylpyrocarbonate (DEPC) water were used to wash the precipitate. After centrifugation at 12,000 g and 4°C for 5 min and evaporation at room temperature (10 min), the precipitate was dissolved with 40 *μ*L of DEPC water (JRDUN Biotechnology) and stored in a refrigerator at –80°C for later assays. For detecting the expression of miRNAs and analyzing the expression of the genes of CUG triplet repeat-binding protein 2 (CUGBP2), cytoplasmic polyadenylation element-binding protein 4 (CPEB4), ER degradation enhancing alpha-mannosidase-like protein 3 (EDEM3), and activating transcription factor 3 (ATF3), PrimeScript RT Master Mix (Fermentas) was used. Then, qPCR assays were subsequently performed in triplicate via SYBR Green qPCR Master Mix (Thermo). The primers are listed in Table 1. GAPDH and U6 were used as the internal references for quantifying mRNA and miRNA, respectively, and the 2^–*ΔΔ*Ct^ method was used to determine their relative expression levels.

### 2.10. Western Blotting Analysis

For extracting the total proteins, a lysate (150–250 *μ*L) containing protease and phosphatase inhibitors (RIPA, Beyotime) was added to the clipped tissue for every 20 mg tissues. The pyrolysis samples were centrifuged at 12,000 × *g* and 4°C for 15 min to obtain the supernatant. The cells were washed with precooled PBS twice after removing the culture medium and then treated with the RIPA protein extraction reagent (Beyotime). The cells were fully lysed at 4°C and scraped into 1.5 mL EP tubes, heated at 95°C for 10 min, and centrifuged at 12,000 × *g* for 10 min; finally, the supernatant was collected. The obtained protein was quantified using the bicinchoninic acid (BCA) protein assay kit (Beyotime), following the manufacturer's instructions, and the extracted proteins were separated by sodium dodecyl-sulfate polyacrylamide gel electrophoresis (SDS-PAGE) with a running buffer. The separated proteins were transferred onto a nitrocellulose membrane using a transfer buffer. Following blockage of the membranes with 5% nonfat milk for 1 h or overnight at 4°C, incubation was performed with primary antibodies against alpha-smooth muscle actin (*α*-SMA, 1 : 1000, Abcam, Ab5694), CHOP (1 : 200, Abcam, Ab11419), glucose-regulated protein 78 (GRP78, 1 : 1000, Abcam, Ab108615), small mother against decapentaplegic 2/3 (Smad2/3, 1 : 1000, Abcam, Ab232326), p-Smad2/3 (1 : 500, Abcam, Ab272332), CUGBP2 (1 : 5000, Abcam, Ab186430), CPEB4 (1 : 1000, Invitrogen, PA5-25538), EDEM3 (1 : 1000, Invitrogen, PA5–68189), ATF3 (1 : 1000, Abcam, Ab207434), and GAPDH (1 : 5000, Proteintech, 60004-1-1G) at 4°C overnight. Next, the samples were incubated with rabbit anti-mouse HRP (Beyotime, A0216) or goat anti-rabbit HRP (Beyotime, A0208) at a 1 : 1000 dilution for 1 h at room temperature. The protein expression was normalized against GAPDH.

### 2.11. Statistical Analysis

All data in this study were presented as the mean ± standard deviation (SD). GraphPad Prism 8.0 (GraphPad Software, USA) was used to analyze the data; differences between groups were determined by Student's *t*-tests, and those among three or more groups were determined by one-way analysis of variance followed by Tukey's post hoc test. All differences among and between groups were considered to be statistically significant at *p* < 0.05.

## 3. Results

### 3.1. HJXJ Intervention Promoted Renal Function of HDN Mice

**Figure 1:**
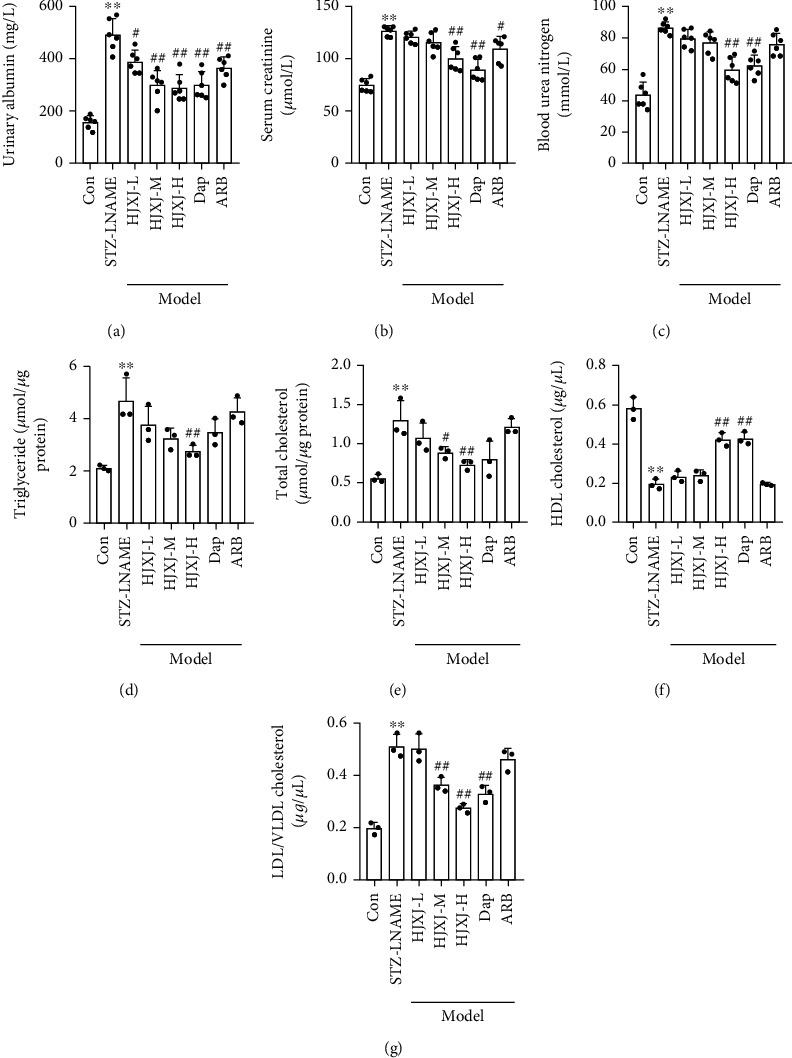
HJXJ intervention improved renal function in HDN mice after 12 weeks. (a) Urinary albumin. (b) Serum creatinine. (c) Blood urea nitrogen. (d) Blood triglycerides. (e) Total cholesterol. (f) High-density lipoprotein (HDL) cholesterol. (g) Low-density lipoprotein (LDL)/very-low-density lipoprotein (VLDL) cholesterol. ^∗∗^*p* < 0.01, vs. Con group; ^#^*p* < 0.05 and ^##^*p* < 0.01, vs. STZ-LNAME group.

Compared to the Con group, STZ-LNAME treatment promoted the level of urinary albumin, serum creatinine, and blood urea nitrogen, whereas a high dose of HJXJ and Dap reduced the concentration of these biochemical molecules (Figures 1(a)–1(c)). Additionally, ARB treatment also decreased the level of urinary albumin and serum creatinine but did not affect the level of blood urea nitrogen. The STZ-LNAME treatment increased the levels of blood triglyceride, total cholesterol, and LDL/VLDL cholesterol but decreased the level of HDL cholesterol (Figures 1(d)–1(g)). However, a high dose of HJXJ effectively reversed the effects of the STZ-LNAME treatment. HJXJ-M also reduced the levels of total cholesterol and LDL/VLDL cholesterol. ARB treatment led to a decrease in the LDL/VLDL cholesterol levels and an increase in the HDL cholesterol levels.

**Figure 2:**
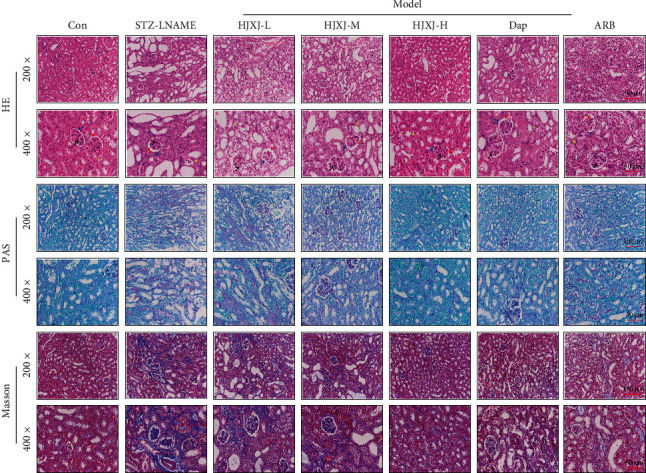
HJXJ intervention ameliorated the pathological changes in HDN mice after 12 weeks. Representative images of kidney tissues stained with H&E; tissue sections were observed at 200x magnification (scale bar = 100 *μ*m) and 400x magnification (scale bar = 50 *μ*m). Black arrowheads indicate glomerulus. Blue arrowheads indicate Bowman capsules. Red arrowheads indicate glomerular space. Yellow asterisks indicate interstitial regions. Representative images of kidney tissues stained with PAS; tissue sections were observed at 200x magnification (scale bar = 100 *μ*m) and 400x magnification (scale bar = 50 *μ*m). Representative images of kidney tissues were stained with Masson; tissue sections were observed at 200x magnification (scale bar = 100 *μ*m) and 400x magnification (scale bar = 50 *μ*m).

We performed H&E staining, PAS staining, and Masson staining to evaluate the pathological changes (Figure 2). In the Con group, intact glomerulus (black arrowheads), Bowman capsules (blue arrowheads), and clear renal parenchymal cells were observed. A series of abnormal appearances were observed in HDN mice, including reduced parenchymal loss, disrupted glomerulus, tubular dilation, mesangial proliferation, basement membrane thickening, and severe fibrosis. Bowman capsules were damaged, and the number of cells in the outer layer of the Bowman's capsule decreased. The glomerular space (red arrowheads) was also destroyed. Additionally, extracellular matrix proteins accumulated in the interstitial regions (yellow asterisks) of the renal tubules. However, HJXJ considerably reduced pathological changes in a dose-dependent manner, and Dap or ARB treatment also showed similar effects.

**Figure 3:**
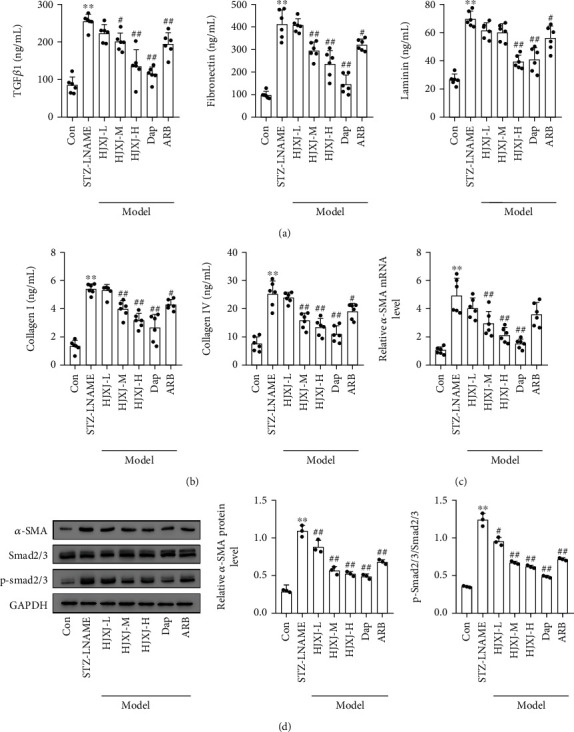
HJXJ intervention reduced fibrosis in STZ-LNAME-induced mice after 12 weeks. (a) ELISA was performed to detect the level of TGF*β*1, fibronectin, and laminin in the serum of HDN mice after 12 weeks of HJXJ intervention. (b) ELISA was performed to detect the level of TGF*β*1, fibronectin, laminin, and collagen (COL I and COL IV) in the serum of HDN mice after 12 weeks of HJXJ intervention. (c) The mRNA level of *α*-SMA in the kidney tissue of HDN mice after 12 weeks of HJXJ intervention was evaluated by RT-qPCR. (d) Western blotting assays were performed to measure the level of *α*-SMA, Smad2/3, and p-smad2/3 in the kidney tissue of HDN mice after 12 weeks of HJXJ intervention. ^∗∗^*p* < 0.01, vs. Con group; ^#^*p* < 0.05 and ^##^*p* < 0.01, vs. STZ-LNAME group.

To determine the effect of HJXJ on the fibrosis of STZ-LNAME mice, we assessed the level of fibrosis molecules after 12 weeks of HJXJ treatment. Compared to Con treatment, STZ-LNAME treatment substantially increased the level of TGF*β*1, fibronectin, laminin, COL I, and COL IV (Figures 3(a) and 3(b)), while HJXJ-M and HJXJ-H intervention decreased the levels of TGF*β*1, fibronectin, Col I, and Col IV; HJXJ-H also significantly decreased the level of laminin. Dap treatment and ARB treatment also reduced the levels of these molecules. Additionally, HJXJ-M, HJXJ-H, and Dap treatment decreased the level of *α*-SMA (mRNA and protein level) and p-smad2/3 in the kidney tissues of mice administered with STZ-LNAME (Figures 3(c) and 3(d)). HJXJ-L and ARB treatment decreased the levels of *α*-SMA protein and p-smad2/3 but did not affect the mRNA level of *α*-SMA.

### 3.2. HJXJ Intervention Reduced ER Stress and the Expression of the Downstream Molecules in HDN Mice

**Figure 4:**
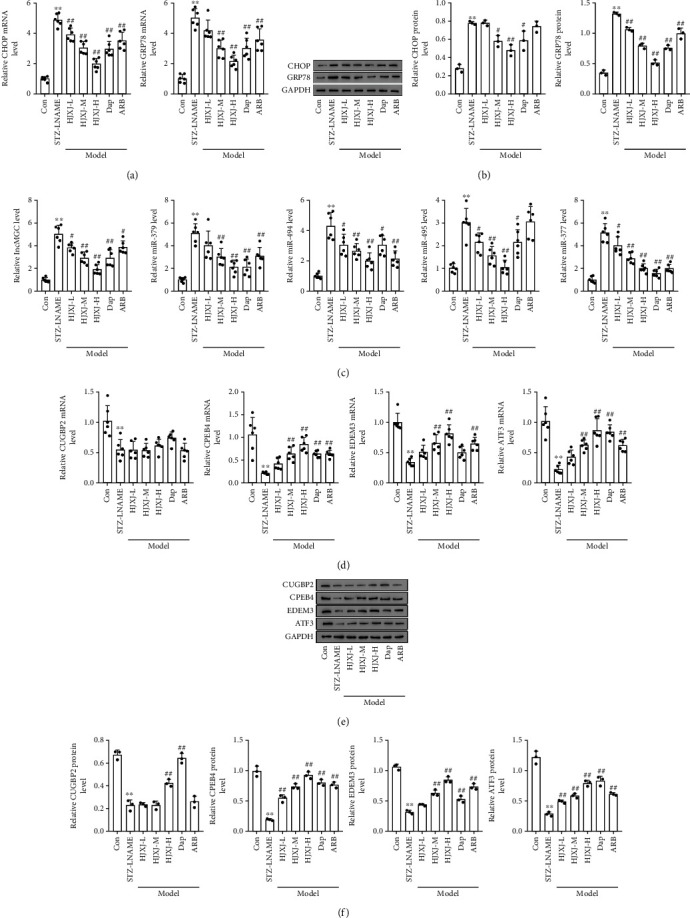
HJXJ intervention decreased ER stress and the expression of the downstream molecules after STZ-LNAME induction. (a) The mRNA levels of the ER stress chaperone genes (*CHOP* and *GRP78*) were detected by RT-qPCR. (b) The protein levels of CHOP and GRP78 were estimated by Western blotting assays. (c) RT-qPCR analysis was performed to detect the expression of ER stress-induced *lncMGC*, *miR379*, *miR494*, *miR495*, and *miR377*. (d) The mRNA levels of *CUGBP2*, *CPEB4*, *EDEM3*, and *ATF3* were detected by RT-qPCR analysis. (e) The protein levels of CUGBP2, CPEB4, EDEM3, and ATF3 were estimated by Western blotting assays. (f) The histogram analysis of the grey intensity of CUGBP2, CPEB4, EDEM3, and ATF3. ^∗∗^*p* < 0.01, vs. Con group; ^#^*p* < 0.05 and ^##^*p* < 0.01, vs. STZ-LNAME group.

To determine the effect of HJXJ on the ER stress of HDN mice, we evaluated the expression of CHOP and glucose-regulated protein 78 (GRP78) after administering HJXJ for 12 weeks. Compared to the mice in the Con group, the HDN mice had higher mRNA and protein levels of CHOP and GRP78 (Figures 4(a) and 4(b)). Treatment with HJXJ decreased the levels of CHOP and GRP78 in a dose-dependent manner, and Dap or ARB treatment also decreased the expression of CHOP and GRP78. The expression of ER stress-induced lncMGC, miR379, miR494, miR495, and miR377 also increased in HDN mice compared to that in the Con mice (Figure 4(c)). HJXJ treatment decreased the levels of lncMGC, miR379, miR494, miR495, and miR377 in a dose-dependent manner. Dap treatment also decreased the expression of lncMGC and the above-mentioned miRNAs. ARB downregulated the expression of lncMGC, miR379, miR494, and miR377.

The targeted expression of lncMGC and these miRNAs, including CUGBP2, CPEB4, EDEM3, and ATF3, decreased at the mRNA and protein level in the STZ-LNAME-treated mice compared to their expression levels in the Con mice (Figures 4(d)–4(f)). Only HJXJ-H and Dap treatments increased the protein level of CUGBP2; however, the other treatments did not affect the expression of CUGBP2. Treatment with HJXJ-M, HJXJ-H, Dap, and ARB increased the mRNA and protein levels of CPEB4 and ATF3. HJXJ-L treatment only increased the expression of CPEB4 and ATF3 at the protein level. Additionally, treatment with HJXJ-M, HJXJ-H, and ARB increased the mRNA and protein levels of EDEM3, but Dap treatment only increased the expression of EDEM3 at the protein level; HJXJ-L treatment did not affect the expression of EDEM3.

### 3.3. HJXJ Treatment Decreased the Level of Fibrosis Molecules in Model HGMCs

We evaluated the *in vitro* toxicity of different concentrations of HJXJ (0 mg/mL, 0.02 mg/mL, 0.05 mg/mL, 0.1 mg/mL, 0.2 mg/mL, 0.5 mg/mL, and 1 mg/mL) on the HGMC and HK-2 cell lines (Figure S1). Treatment with HJXJ did not affect the viability of HGMCs after 24 h and 48 h of treatment. HJXJ treatment had a similar effect on HK-2 cells as it had on HGMCs after 24 h of treatment. However, after 48 h of treatment, 0.1 mg/mL, 0.2 mg/mL, and 0.5 mg/mL HJXJ increased the viability of HK-2 cells. After 72 h of treatment, 0.05 mg/mL, 0.1 mg/mL, and 0.2 mg/mL HJXJ increased the viability of HGMC and HK-2 cells. Additionally, 0.5 mg/mL HJXJ also increased the viability of HK-2 cells. Only 1 mg/mL HJXJ decreased the viability of HGMC and HK-2 cells after 72 h of treatment. These results indicated that HJXJ had negligible toxic effects on cells.

**Figure 5:**
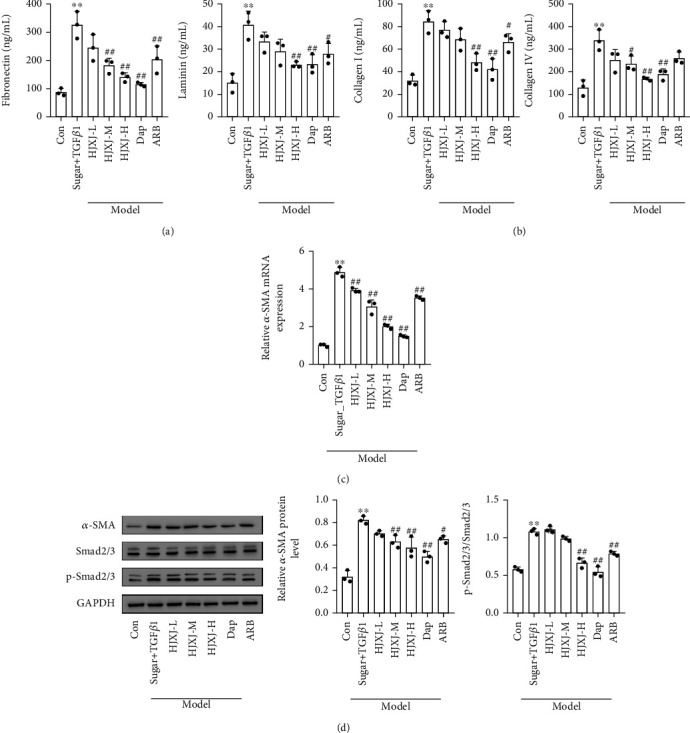
HJXJ treatment decreased the level of fibrosis molecules in HGMCs (model HGMC) induced by high doses of sugar (30 mmol/mL) and TGF*β*1 (5 ng/mL). (a) ELISA was performed to assess the level of fibronectin and laminin. (b) ELISA was performed to assess the collagen (COL I and COL IV) content. (c) The mRNA level of *α-SMA* was evaluated by RT-qPCR analysis. (d) Western blotting assays were performed to measure the levels of *α*-SMA, Smad2/3, and p-smad2/3. ^∗∗^*p* < 0.01, vs. Con group; ^#^*p* < 0.05 and ^##^*p* < 0.01, vs. sugar+TGF*β*1 group.

To determine the effect of HJXJ on the fibrosis of HGMCs (model HGMC) induced by high doses of sugar (30 mmol/mL) and TGF*β*1 (5 ng/mL), we assessed the level of fibrosis molecules after treatment with HJXJ. The concentration of fibronectin, laminin, COL I, and COL IV increased after high doses of sugar and TGF*β*1 were administered (Figures 5(a) and 5(b)). Treatment with HJXJ-H and Dap decreased the levels of these molecules. HJXJ-M decreased the content of fibronectin and COL IV, and ARB treatment decreased the levels of fibronectin and laminin. The high doses of sugar and TGF*β*1 also promoted the expression of *α*-SMA and the level of p-Smad2/3. HJXJ-M, HJXJ-H, Dap, and ARB treatment decreased the level of *α*-SMA (mRNA and protein levels) in model HGMCs (Figures 5(c) and 5(d)). HJXJ-H, Dap, and ARB treatment also decreased the level of p-Smad2/3.

### 3.4. HJXJ Intervention Reduced the ER Stress and the Expression of the Downstream Molecules in the Model HGMCs

**Figure 6:**
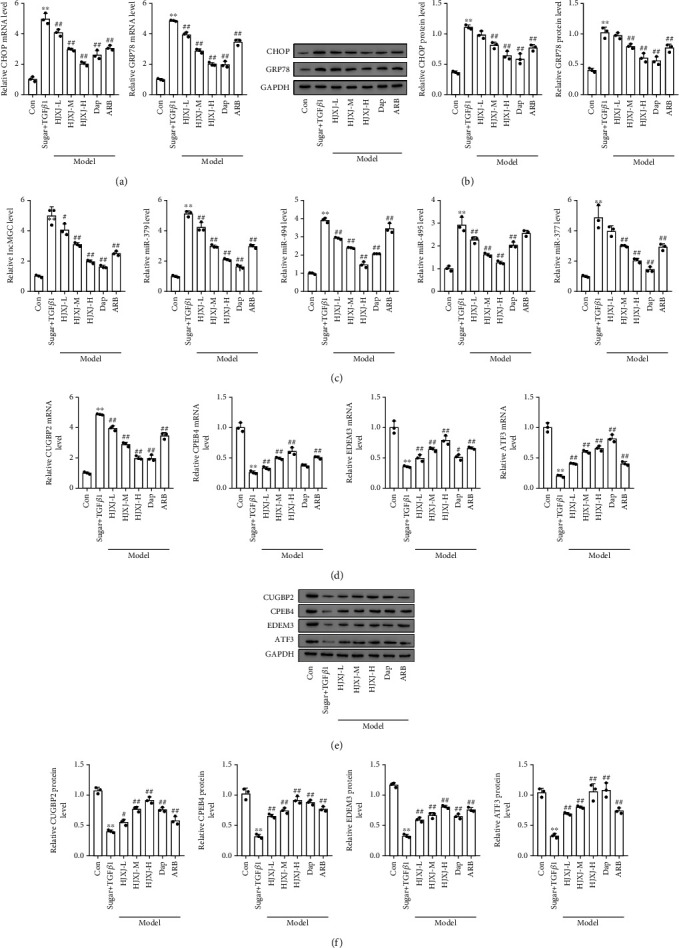
HJXJ intervention decreased ER stress and the expression of the downstream molecules in HGMCs induced by high doses of sugar (30 mmol/mL) and TGF*β*1 (5 ng/mL). (a) The mRNA levels of ER stress chaperone genes (*CHOP* and *GRP78*) were detected by RT-qPCR analysis. (b) The protein levels of CHOP and GRP78 were measured by Western blotting assays. (c) RT-qPCR was performed to evaluate the expression of ER stress-induced *lncMGC*, *miR379*, *miR494*, *miR495*, and *miR377*. (d) The mRNA levels of *CUGBP2*, *CPEB4*, *EDEM3*, and *ATF3* were detected by RT-qPCR. (e) The protein levels of CUGBP2, CPEB4, EDEM3, and ATF3 were estimated by Western blotting assays. (f) The histogram analysis of the grey intensity of CUGBP2, CPEB4, EDEM3, and ATF3 was performed. ^∗∗^*p* < 0.01, vs. Con group; ^#^*p* < 0.05 and ^##^*p* < 0.01, vs. the STZ-L-NAME group.

We investigated the effect of HJXJ on the ER stress in HGMCs after treatment with high doses of sugar and TGF*β*1. The expression of CHOP and GRP78 in the above-mentioned HGMCs increased at the mRNA and protein levels (Figures 6(a) and 6(b)). HJXJ-M, HJXJ-H, Dap, and ARB decreased the levels of CHOP and GRP78, but HJXJ-L only decreased the mRNA level of CHOP and GRP78. In model HGMCs, the expressions of lncMGC, miR379, miR494, miR495, and miR377 were upregulated, while HJXJ-M, HJXJ-H, Dap, and ARB decreased the levels of these molecules (Figure 6(c)). The mRNA and protein levels of CUGBP2, CPEB4, EDEM3, and ATF3 were lower in model HGMCs compared to those in control HGMCs (Figures 6(d)–6(f)). HJXJ increased the protein and mRNA levels of CUGBP2, CPEB4, EDEM3, and ATF3 in a dose-dependent manner. Dap and ARB also increased the protein and mRNA levels of CUGBP2, CPEB4, EDEM3, and ATF3.

### 3.5. Overexpression of *CHOP* or *lncMGC* Decreased the Effects of HJXJ-M on the Fibrosis Molecules and Downstream Target Molecules in Model HGMCs

**Figure 7:**
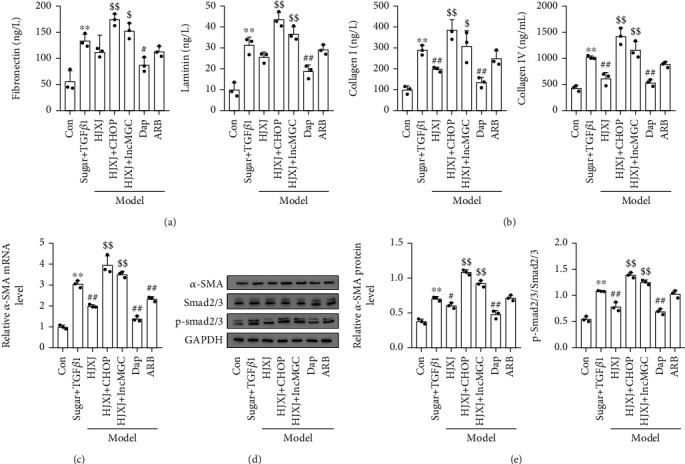
The overexpression of *CHOP* or *lncMGC* decreased the effect of HJXJ-M on fibrosis in HGMCs induced by high doses of sugar (30 mmol/mL) and TGF*β*1 (5 ng/mL). (a) ELISA was performed to detect the levels of fibronectin and laminin in HGMCs. (b) ELISA was performed to determine the level of collagen (Col I and Col IV) in HGMCs. (c) The mRNA level of *α-SMA* in HGMCs was evaluated by RT-qPCR. (d) Western blotting analysis was performed to estimate the level of *α*-SMA, Smad2/3, and p-smad2/3 in HGMCs. (e) The histogram analysis of the grey intensity of *α*-SMA, Smad2/3, and p-smad2/3 in HGMCs. ^∗∗^*p* < 0.01, vs. Con group; ^#^*p* < 0.05 and ^##^*p* < 0.01, vs. sugar+TGF*β*1 group; ^$^*p* < 0.05 and ^$$^*p* < 0.01, vs. HJXJ group.

We constructed the HGMC model, in which *CHOP* or *lncMGC* was overexpressed (Figure S2), to understand how CHOP and lncMGC regulate the effects of HJXJ-M on the level of fibrosis molecules after treatment with high doses of sugar (30 mmol/mL) and TGF*β*1 (5 ng/mL). The concentrations of fibronectin, laminin, COL I, and COL IV increased after high doses of sugar and TGF*β*1 were administered (Figures 7(a) and 7(b)). HJXJ-M (HJXJ-M will be shortened to HJXJ in the following description) did not affect the concentration of fibronectin and laminin, but it decreased the content of COL I and COL IV. Compared to HJXJ treatment, *CHOP* or *lncMGC* overexpression increased the concentration of these molecules. HJXJ also decreased the expression of *α*-SMA and the level of p-Smad2/3, while *CHOP* or *lncMGC* overexpression eliminated the effects of HJXJ (Figures 7(c) and 7(d)).

**Figure 8:**
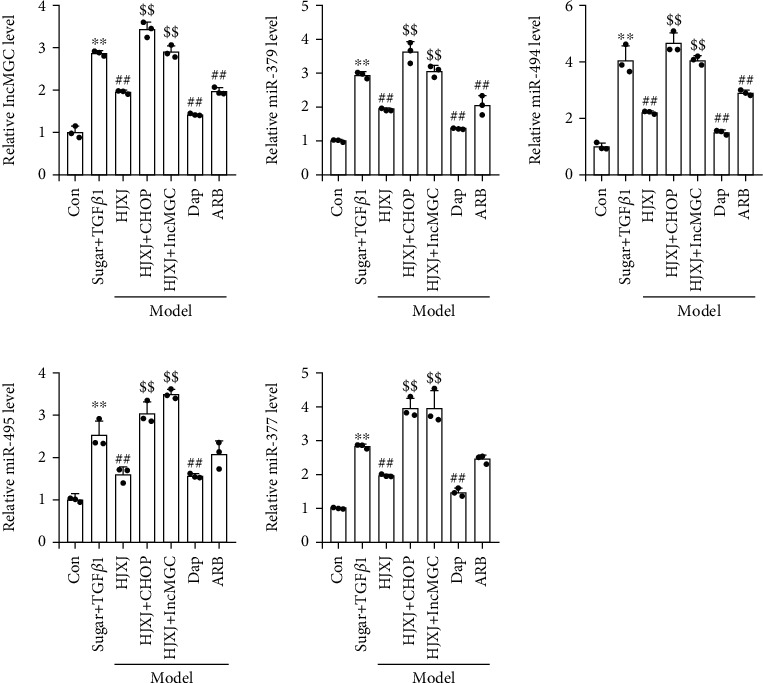
The overexpression of *CHOP* or *lncMGC* regulated the effects of HJXJ on the levels of lncMGC, miR379, miR494, miR495, and miR377 in HGMCs after treatment with high doses of sugar (30 mmol/mL) and TGF*β*1 (5 ng/mL). RT-qPCR was performed to detect the expression of ER stress-induced *lncMGC*, *miR379*, *miR494*, *miR495*, and *miR377*. ^∗∗^*p* < 0.01, vs. Con group; ^##^*p* < 0.01, vs. sugar+TGF*β*1 group; ^$$^*p* < 0.01, vs. HJXJ group.

**Figure 9:**
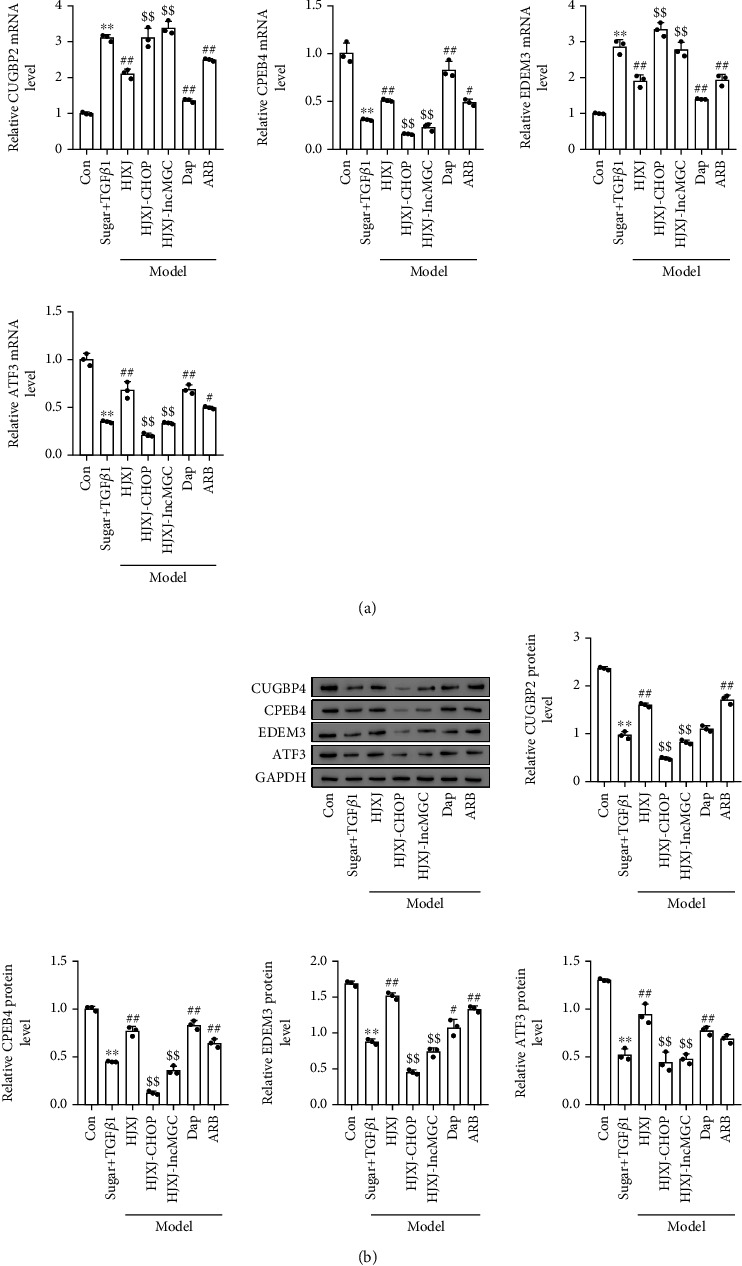
The overexpression of *CHOP* or *lncMGC* regulated the effects of HJXJ on the expression of CUGBP2, CPEB4, EDEM3, and ATF3 in HGMCs after treatment with high doses of sugar (30 mmol/mL) and TGF*β*1 (5 ng/mL). (a) The mRNA levels of *CUGBP2*, *CPEB4*, *EDEM3*, and *ATF3* were detected by RT-qPCR. (b) The protein levels of CUGBP2, CPEB4, EDEM3, and ATF3 were measured by Western blotting assays. ^∗∗^*p* < 0.01, vs. Con group; ^##^*p* < 0.01, vs. sugar+TGF*β*1 group; ^$$^*p* < 0.01, vs. HJXJ group.

The overexpression of *CHOP* or *lncMGC* increased the expression of lncMGC, miR379, miR494, miR495, and miR377; however, their expression decreased after HJXJ was administered (Figure 8). Compared to the model HGMC, HJXJ treatment upregulated the levels of CUGBP2, CPEB4, EDEM3, and ATF3. However, these effects were eliminated after *CHOP* or *lncMGC* was overexpressed (Figure 9).

## 4. Discussion

In this study, we investigated the curative effect of HJXJ in hypertensive diabetic nephropathy and its molecular mechanism. We constructed HDN mouse models by administering STZ and LNAME. For studying the pathophysiology of diabetes mellitus, STZ is widely used to induce diabetic nephropathy; STZ also effectively induces renal injury in the diabetic animal model. Prolonged hyperglycemia destroys the function and structure of the kidneys [20]. LNAME, the NO synthase inhibitor, decreases the NO synthase activity of the heart, kidneys, aorta, and brain to induce the “NO-deficient” model of hypertension [21, 22]. In this study, the combined administration of STZ and LNAME increased renal injury in mice; for example, it increased the concentration of urinary albumin, serum creatinine, blood urea nitrogen, blood triglyceride, total cholesterol, and LDL/VLDL cholesterol and decreased HDL cholesterol levels. However, the HJXJ treatment reversed the effects of the STZ-LNAME treatment. These measured indices also characterized the effect of HJXJ on the alterations in lipid and protein metabolism of HDN mice. The improvement of diabetic nephropathy involves the transformation of moderate or severe albuminuria into normal or mildly increased albuminuria [23]. Serum creatinine is used to evaluate renal function [24]. An increase in the level of triglycerides and a decrease in the level of HDL cholesterol are the characteristics of dyslipidemias in patients with diabetes [25]. Low levels of HDL cholesterol and high levels of triglycerides are independent risk factors for DKD [26]. In the early stage of DKD, the pathological features include glomerular hypertrophy, thickened basement membrane, and glomerulosclerosis, whereas the late stage is characterized by tubular atrophy and interstitial fibrosis, which result in a loss of renal function [27, 28]. In DKD, excessive deposition of extracellular matrix commonly occurs in the glomeruli and renal tubules in DN, causing glomerulosclerosis and renal interstitial fibrosis [29]. In our study, STZ-LNAME induced a series of abnormal appearances in the renal tissue of mice, but HJXJ treatment reversed the effects.

To evaluate the changes in the animal model, urinary creatinine levels were assessed in the control group and the model group. However, the differences between the groups were not significant. Serum creatinine is produced by nonenzymatic anhydration of creatine in muscle cells [30]. In healthy individuals, creatinine is primarily removed by the kidneys. In CKD patients, a decrease in the excretion of urinary creatinine is associated with a higher risk of kidney failure and mortality [31]. Some studies have suggested that in cases of kidney insufficiency, other pathways might be involved in the degradation and elimination of serum creatinine. Some researchers have proposed *in vitro* degradation pathways for creatinine [32, 33]. When the concentration of serum creatinine increases, a larger amount of creatinine is excreted in the bowel, where bacterial creatininase converts it back to creatine, which is then partially reabsorbed by the gut through the enteric cycle [33]. Therefore, the detection of serum creatinine is a better technique than assessing the concentration of urinary creatinine for evaluating the therapeutic effect of HJXJ on HDN mice. Many studies have also evaluated kidney diseases by measuring serum creatinine [34, 35].

In this study, Dap and ARB were used as positive control drugs for treating hypertensive diabetic nephropathy. Dap is an oral sodium-glucose cotransporter type 2 inhibitor, and it is used as a second or third-line antihyperglycemic treatment option for patients with type 2 diabetes. It was approved by the European Medicines Agency and the US Food and Drug Administration [36]. A double-blind and randomized trial was performed to investigate the effects of Dap on kidney and cardiovascular diseases in the presence or absence of type 2 diabetes. The results showed that Dap decreased the risk of polycystic kidney disease and cardiovascular events in patients with diabetic and nondiabetic chronic nephropathy [37]. In hyperglycemia-induced mice, Dap reduced the expression of TGF*β*1, fibronectin, and COL IV to decrease the progression of diabetes-associated glomerulosclerosis [38]. ARB is an angiotensin receptor blocker, which is widely used for treating hypertension and has renoprotective effects. It can decrease proteinuria in patients with diabetic and nondiabetic nephropathy [39]. In a multicenter and double-blind study on 885 hypertensive patients with type 2 diabetes, ARB regulated blood pressure control, and in patients with better blood pressure control, ARB had greater renoprotective effects [40]. In this study, Dap and ARB considerably improved the renal function of the STZ-LNAME-induced mice.

Several studies have reported that the ER stress response is activated in diabetic hypertensive nephropathy. ERS responds adaptively to cellular damage, such as cellular calcium imbalance, hypoxia, glucose starvation, and excessive accumulation of unfolded protein [41–43]. ER stress leads to the activation of x-box binding protein 1, ATF3/ATF4, and ATF6, and these transcription factors increase the expression of other transcription factors, such as CHOP and GRP78 [44, 45]. In various noninflammatory and inflammatory glomerulopathy, many ERS markers of glomeruli are identified by human kidney biopsies [46]. CHOP is a well-known marker of ERS, and as a transcription factor, it is associated with apoptosis. Its biological effects depend on its heterodimeric partner or the phosphorylation status of eIF2*α* [47–50]. In diabetic mice induced with streptozotocin, ERS-mediated upregulation of CHOP was found in the kidneys of diabetic mice [51]. GRP78 facilitates the proper folding of de novo proteins and regulates the unfolded protein response to control ERS in the ER [52]. In Kunming mice, a combination treatment of a high-fat diet with STZ was found to upregulate the expression of CHOP and GRP78 in the kidneys [53]. In our study, an increase in the expression of CHOP and GRP78 was observed in mice induced by STZ-LNAME and model HGMCs, but HJXJ treatment decreased the expression of CHOP and GRP78. Additionally, the positive control drugs (Dap and ARB) also decreased the levels of CHOP and GRP78. In another study, ARB was found to decrease the expression of ERS markers (CHOP and XBP-1) and the activation of profibrotic growth factors, such as TGF*β*, in mice with STZ-induced diabetic kidney disease [54]. Therefore, the inhibition of ERS might be an effective method for improving hypertensive diabetic nephropathy. Some studies have shown that ATF3 enhanced the transcription of CHOP [55, 56]. ATF3 can suppress the transcription of the *CHOP* gene, and *CHOP* can repress the function of the ATF3 protein [57]. In our study, the overexpression of *CHOP* decreased the expression of ATF3, which was probably mediated by an increase in the expression of miR494. Another study found that at the matrix metalloproteinase 13 (MMP13) promoter, ATF3 and Smad4 formed a complex to regulate the expression of MMP13 after treatment with TGF-*β*1 [58].

The deletion of the miR379 cluster attenuates hepatic steatosis and metabolic dysfunction of leptin-receptor-deficient type 2 mice induced by a high-fat diet, and the insulin-like growth factor 1 receptor is directly targeted by miR379 [59]. In a study, ERS upregulated the megacluster miR379 along with lncMGC in the kidneys of diabetic mice and induced hypertrophy and fibrosis in mice. Also, the deletion of *CHOP* reduced the expression of the cluster miR379 and lncMGC [11]. STZ significantly increases the expression of miR379, miR377, and miR495, and the reduction of miR379 or its host lncMGC decreases the level of albuminuria and glomerular hypertrophy [60]. In this study, in mice induced by STZ-LNAME and model HGMCs, the expression of lncMGC and the megacluster miR379, including miR379, miR494, miR495, and miR377, increased considerably, while HJXJ treatment decreased the level of these molecules. A study showed that CUGBP2, CPEB4, EDEM3, and ATF3 are the targets of the cluster miR379 [11]. In our study, the targeted molecules of the cluster miR379 increased after HJXJ treatment, and the overexpression of *CHOP* or *lncMGC* decreased the effects of HJXJ treatment. Therefore, HJXJ treatment may decrease hypertensive diabetic nephropathy by regulating the expression of the megacluster miR379 and lncMGC to mediate the regulation of CUGBP2, CPEB4, EDEM3, and ATF3. Another study also reported that miR494 binds to the 3′UTR of ATF3 to decrease the expression of ATF3, leading to adhesion molecule-induced kidney injury in the ischemia/reperfusion model [61].

The extracellular matrix plays an important role in maintaining the structure and development of tissues, and some components of the extracellular matrix, such as fibronectin, laminin, and collagen, form the tubular basement membrane of the kidneys [29]. However, the excessive accumulation and deposition of extracellular matrix proteins cause progressive glomerular and tubulointerstitial fibrosis [62, 63]. Renal interstitial fibrosis plays a key role in all types of progressive chronic kidney disease [64]. In our study, the levels of fibrosis molecules, such as TGF*β*1, fibronectin, laminin, and COL I, increased after treatment with STZ-LNAME, whereas HJXJ decreased the concentration of these molecules. Additionally, sugar and TGF*β*1 promoted the levels of fibronectin, laminin, COL I, and COL IV in HGMCs. TGF-*β* was considerably upregulated in the injured kidney [65, 66]. In renal tubular epithelial cells, TGF-*β*1 induces epithelial-mesenchymal transition, and it is overexpressed in diabetic nephropathy [67, 68]. TGF-*β*1 is the main driver of progressive renal fibrosis in individuals with hypertension, but the mechanism is complex. TGF-*β*1 strongly affects p53 activity to facilitate the p53-Smad2/3 complexes. These complexes bind to the promoter of the profibrotic gene, such as the plasminogen activator inhibitor-1 gene [69]. Another study showed that TGF-*β*1 activated activin receptor-like kinase 5 (ALK5) to phosphorylate Smad2/3, which drives the fibrotic process [70]. The phosphorylated Smad2 enters the nucleus and induces the secretion of COL I and COL IV and an increase in the level of the *α*-SMA protein in renal tubular epithelial cells [71, 72].

**Figure 10:**
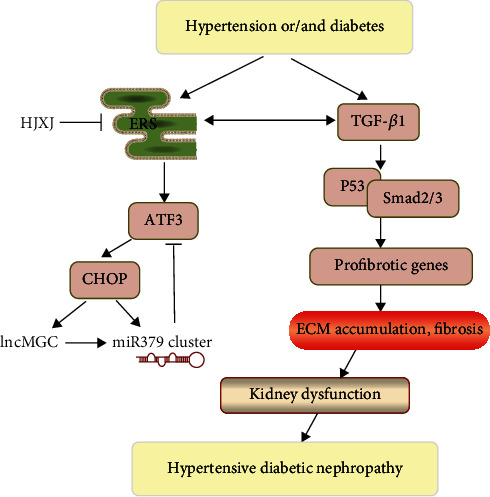
The Huaju Xiaoji formula influences ERS-lncMGC/miRNA to regulate the extracellular matrix proteins in hypertensive diabetic nephropathy.

A study found that TGF-*β*1 induced the expression of ERS markers and collagen contraction in fibroblasts and organ cultures [73]. Some studies have shown that ERS greatly contributes to hypertension and hypertension-related pathology [74, 75]. The ERS inhibitor tauroursodeoxycholic acid significantly reduces systolic blood pressure, suggesting that ERS can promote an increase in blood pressure [76–78]. ERS influences heart damage by increasing apoptosis and fibrosis in hypertensive mice [79]. The alterations of extracellular matrix proteins change the composition of the aortic wall, leading to vascular fibrosis and dysfunction in hypertension [80, 81]. The aggregation of extracellular matrix proteins also promotes chronic tissue injury [82]. ERS triggers collagen synthesis in osteoblasts and gingival fibroblasts [83, 84]. Mice with a genetic defect in the ER chaperone protein show impaired function of collagen synthesis [85]. In an Ang II-induced rat hypertension model, ERS induction increased aortic collagen content, fibrosis, and MMP-2 activity, whereas the inhibition of ER stress decreased blood pressure, collagen content, and fibrosis, while improving vascular functions [86]. In this study, we found that the overexpression of *CHOP* or *lncMGC* increased the expression of extracellular matrix proteins, including fibronectin, laminin, COL I, and COL IV, in HGMCs. The reduction in the level of miR379 resulted in a decrease in the accumulation of the extracellular matrix [60]. Therefore, we speculated that ERS-lncMGC/miRNA might affect the course of hypertensive diabetic nephropathy by altering the composition and content of the extracellular matrix, and TGF-*β*1 might play an important role in these processes (Figure 10).

Traditional Chinese medicine is extensively used in clinical settings due to its minimal toxicity and low side effects [87]. In a clinical study, it was found that the Yiqi Huaju Qingli formula had a positive effect on patients with metabolic syndrome. Additionally, no known side effects were observed during routine liver and kidney hematuria analysis of patients [16]. HJXJ was developed based on the Yiqi Huaju Qingli formula. Additionally, HJXJ had negligible toxic effects on HGMC and HK-2 cells *in vitro*. Therefore, HJXJ might also have minimal toxicity *in vivo*. However, to provide better guidance for the clinical application of HJXJ, future studies need to examine and assess its safety by isolating various organs, such as the small intestine, stomach, and liver, after administering HJXJ to mice.

However, our study had some limitations. The size of the animal sample was small; thus, future studies need to increase the sample size to confirm the curative effect of HJXJ on hypertensive diabetic mice with nephropathy. Because ERS can trigger apoptotic signals, the apoptosis of model HGMCs regulated by HJXJ treatment needs to be further investigated. We only showed that HJXJ treatment downregulated the expression of CHOP and GRP78 in HDN mice and model HGMCs, but the direct targets of HJXJ that regulate the expression of CHOP and GRP78 need to be assessed in the future. We only confirmed that HJXJ treatment upregulated the expression of CUGBP2, CPEB4, EDEM3, and ATF3. In the future, we need to determine the mechanisms by which these molecules influence the biological behaviors of HGMCs after treatment with HJXJ. Additionally, HJXJ contains various active ingredients from multiple traditional Chinese medicines. Therefore, the major active ingredients of HJXJ need to be identified in future studies. We also need to investigate whether these major active ingredients can regulate ERS to improve the condition of hypertensive diabetic mice with nephropathy.

## 5. Conclusion

To summarize, in this study, we proposed that the HJXJ formula might regulate ERS-lncMGC/miRNA to enhance renal function in hypertensive diabetic mice with nephropathy. Our study demonstrated a new mechanism underlying the therapeutic effects of HJXJ on hypertensive diabetic nephropathy, which might help us better understand the pathogenesis of this disease. In this study, we determined the role of HJXJ in an in vitro cell model and assessed the function of HJXJ *in vivo*. However, as the pathogenic mechanism of hypertensive diabetic nephropathy is very complex, further investigation is required.

## Figures and Tables

**Table 1:** Primer sequences used in quantitative real-time polymerase chain reaction (RT-qPCR).

Gene	Forward primer (5′ to 3′)	Reverse primer (5′ to 3′)
CUGBP (mus)	CTATGAACGGCTTTCAGATCGG	GACATGCTAGGCAAACGATGAC
CPEB4 (mus)	CGCAAGCAATCATATTCAG	CATCGGAAACAATGAAGAC
EDEM3 (mus)	AAGATGACTGCCCACTCC	GCCGAGATGCCACAATAC
ATF3 (mus)	GAGTGAAACTGGCTGATG	GGCTGTGGTTATCTTTGG
GAPDH (mus)	CTGCCCAGAACATCATCC	CTCAGATGCCTGCTTCAC
miR379–5p (mus)	GCGCGTGGTAGACTATGGAA	AGTGCAGGGTCCGAGGTATT
miR494–3p (mus)	CGCGTGAAACATACACGGGA	AGTGCAGGGTCCGAGGTATT
miR495–3p (mus)	GCGAAACAAACATGGTGCA	AGTGCAGGGTCCGAGGTATT
miR377–3p (mus)	CGCGATCACACAAAGGCAAC	AGTGCAGGGTCCGAGGTATT
U6 (mus)	GCTTCGGCAGCAC	GGAACGCTTCACG
CUGBP (homo)	CGTGTTGTATTGTTGGTAG	TTACTGAGGTGAACTGTAG
CPEB4 (homo)	TCACTGATTGCCTAAACC	ACACAAACACCCATTACC
EDEM3 (homo)	CTACCAGTGGCCTTCCTTATC	TCAATCCCTGCTCCAACTC
ATF3 (homo)	TACTCTTCCGATGTTTGTG	CTTTCTTCCTGTGACTTTG
GAPDH (homo)	AATCCCATCACCATCTTC	AGGCTGTTGTCATACTTC
miR379–5p (homo)	GCGCGTGGTAGACTATGGAA	AGTGCAGGGTCCGAGGTATT
miR494–3p (homo)	CGCGTGAAACATACACGGGA	AGTGCAGGGTCCGAGGTATT
miR495–3p (homo)	GCGAAACAAACATGGTGCA	AGTGCAGGGTCCGAGGTATT
miR377–3p (homo)	CGCGATCACACAAAGGCAAC	AGTGCAGGGTCCGAGGTATT
U6 (homo)	CTCGCTTCGGCAGCACA	AACGCTTCACGAATTTGCGT

## Data Availability

The data used to support the findings of this study are available from the corresponding authors upon request.

## Supplementary Materials

Supplementary 1 Figure S1: the cell viability of HGMC and HK-2 cells treated with different concentrations of HJXJ (0 mg/mL, 0.02 mg/mL, 0.05 mg/mL, 0.1 mg/mL, 0.2 mg/mL, 0.5 mg/mL, and 1 mg/mL), as estimated by the CCK-8 assay. ^∗^*p* < 0.05 and ^∗∗^*p* < 0.01, vs. 0 mg/mL HJXJ.

Supplementary 2 Figure S2: the verification of *CHOP* or *lncMGC* overexpression in HGMC cells. (A) The protein level of CHOP was measured by western blotting. (B) The expression levels of *CHOP* and *lncMGC* were detected by RT-qPCR. ^∗∗^*p* < 0.01, vs. Con group.
