# Supplementary figures and images for: Huaju Xiaoji Formula Regulates ERS-lncMGC/miRNA to Enhance the Renal Function of Hypertensive Diabetic Mice with Nephropathy
Source: J Diabetes Res. 2024 Jan 20;2024:6942156. doi: 10.1155/2024/6942156 (PMC10821808; doi:10.1155/2024/6942156)

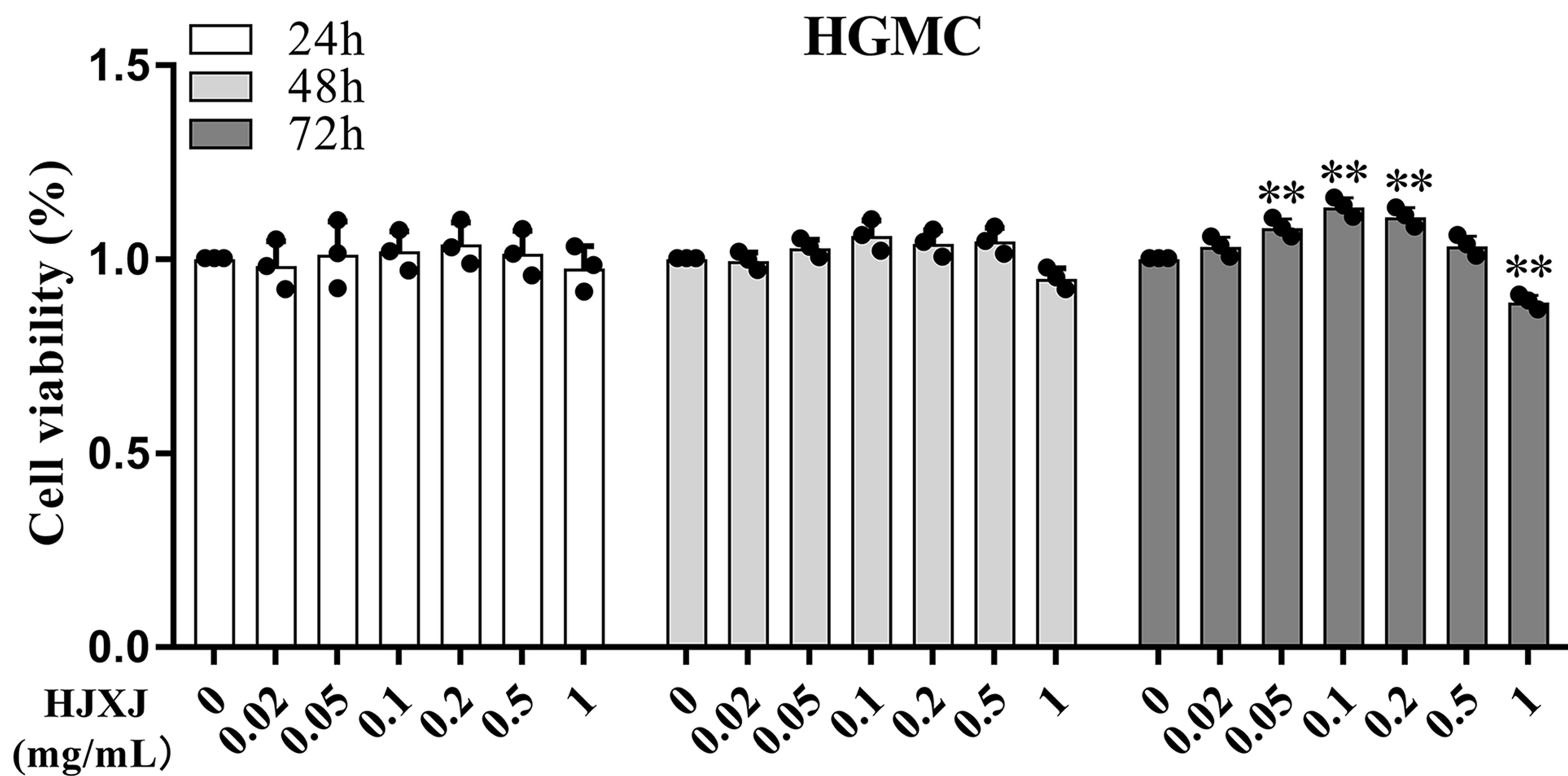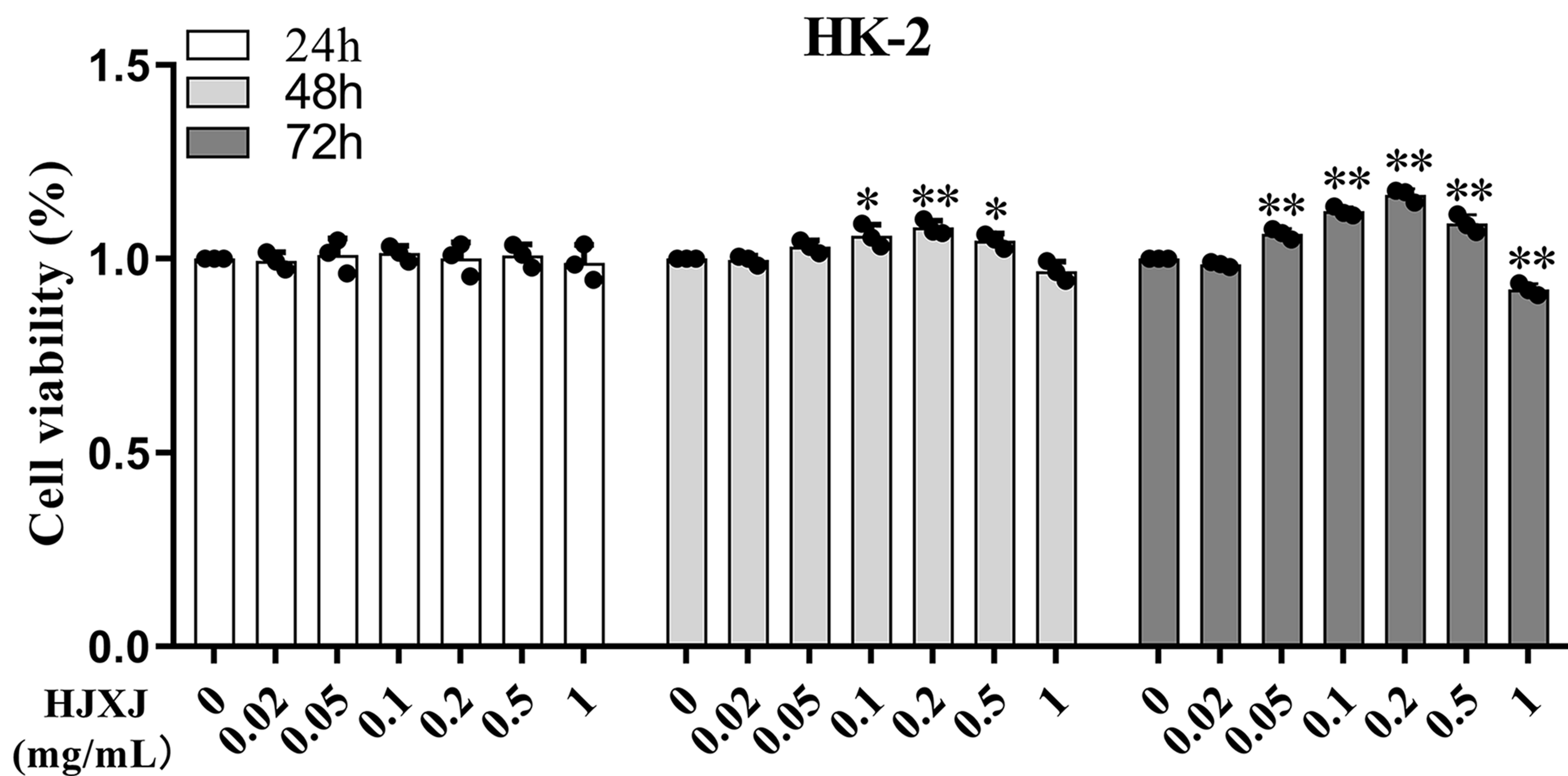

Supplement: Supplementary 1 — Figure S1: the cell viability of HGMC and HK-2 cells treated with different concentrations of HJXJ (0 mg/mL, 0.02 mg/mL, 0.05 mg/mL, 0.1 mg/mL, 0.2 mg/mL, 0.5 mg/mL, and 1 mg/mL), as estimated by the CCK-8 assay. ∗p < 0.05 and ∗∗p < 0.01, vs. 0 mg/mL HJXJ. [file 6942156.f1.pdf]

A

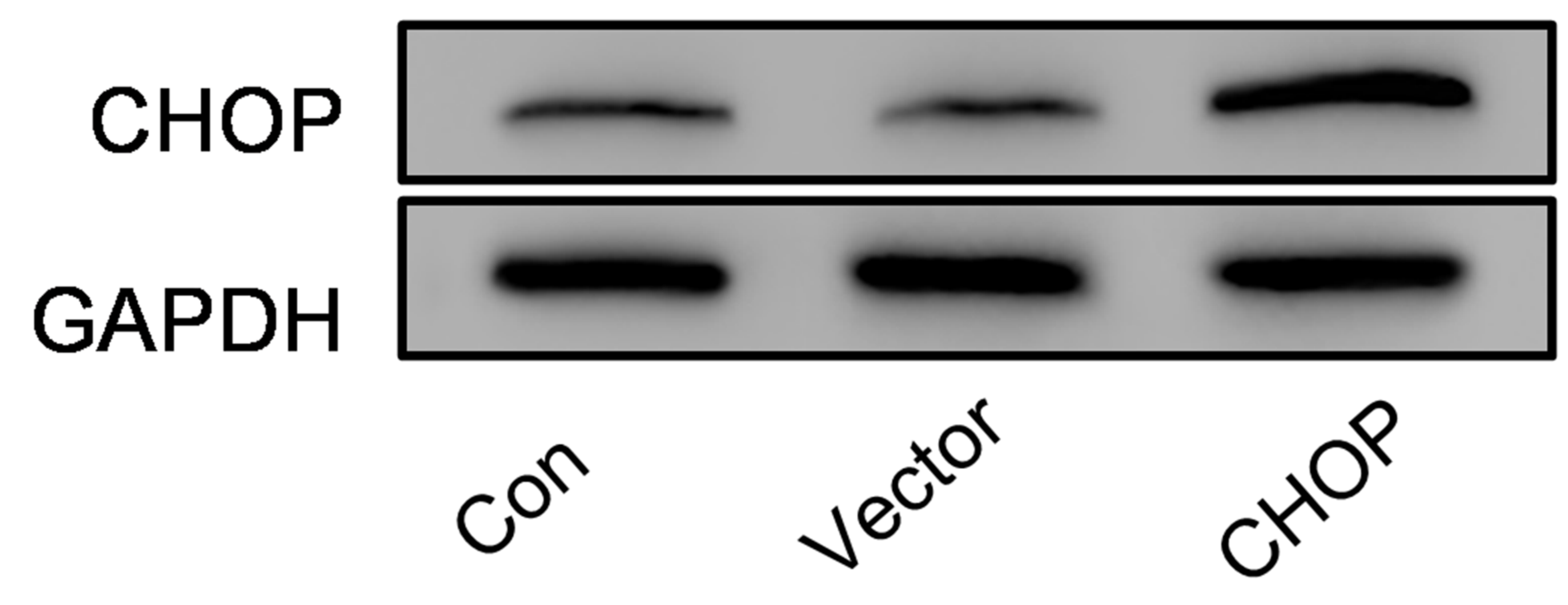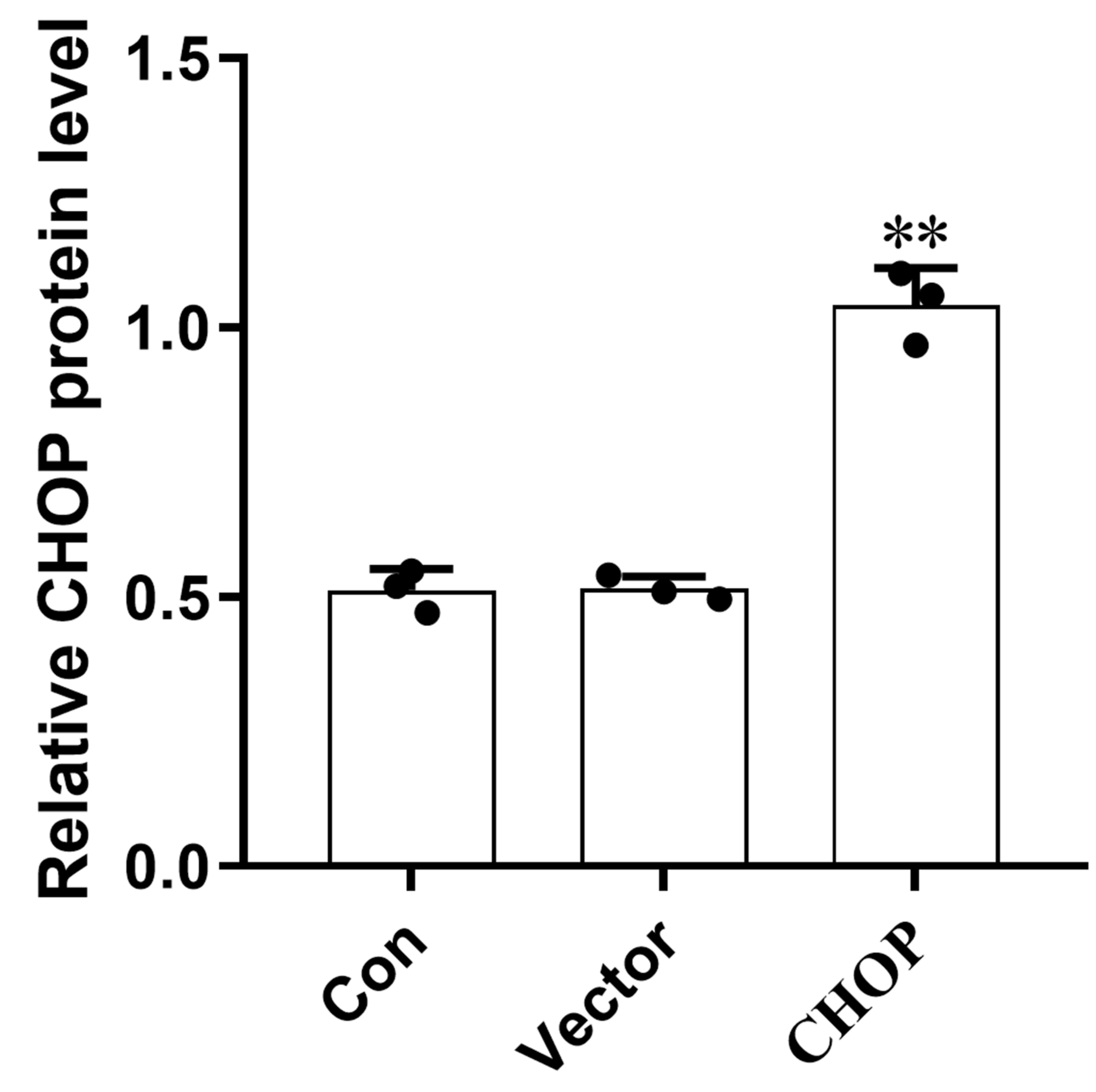

B

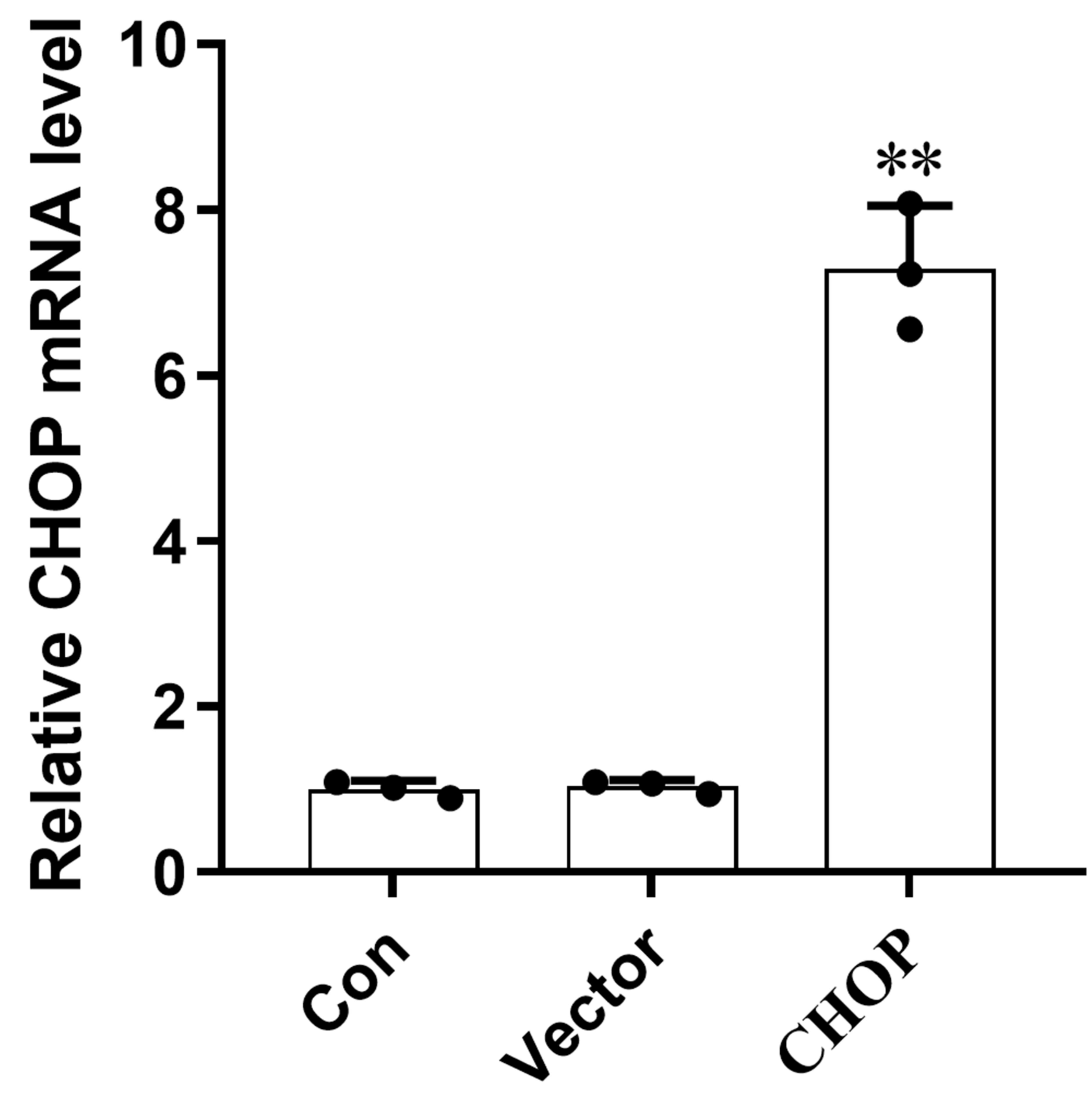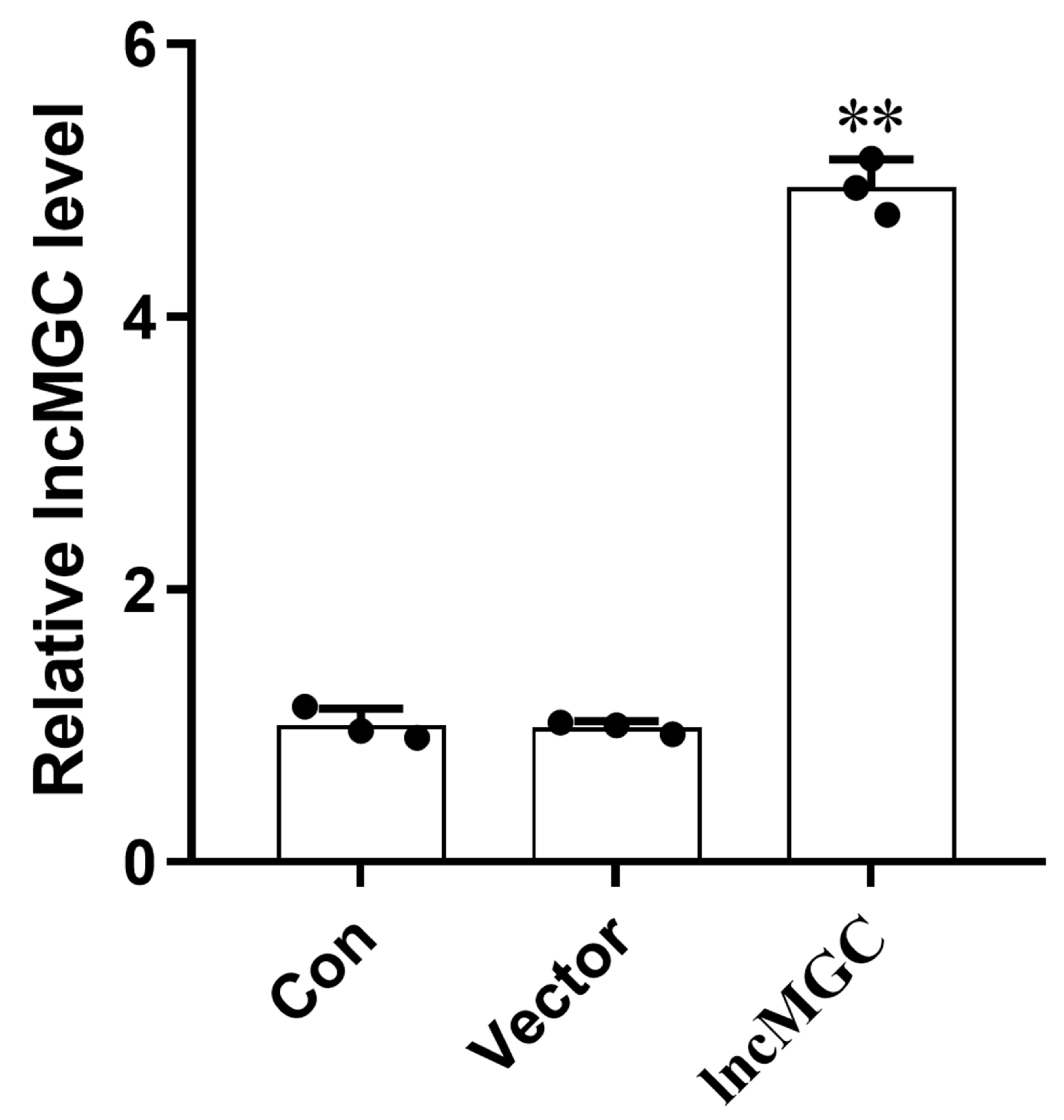

Supplement: Supplementary 2 — Figure S2: the verification of CHOP or lncMGC overexpression in HGMC cells. (A) The protein level of CHOP was measured by western blotting. (B) The expression levels of CHOP and lncMGC were detected by RT-qPCR. ∗∗p < 0.01, vs. Con group. [file 6942156.f2.pdf]
